# Steroid-Responsive Myositis Associated With Acute Hepatitis E Infection

**DOI:** 10.7759/cureus.15492

**Published:** 2021-06-07

**Authors:** Lubna Jafri, Ali Sajjad, Shafaq Saleem, Hira Jameel, Dureshahwar Kanwar

**Affiliations:** 1 Neurology, Aga Khan University, Karachi, PAK

**Keywords:** hepatitis e virus, hev, myopathy, myositis, steroids

## Abstract

Acute hepatitis E virus (HEV) infection is usually self-limiting and presents as mild jaundice accompanied by malaise, anorexia, nausea, vomiting, abdominal pain, or fever. Rarely, it can lead to fulminant hepatic failure especially in pregnant women or cause extrahepatic manifestations. We report a case of a young woman already diagnosed with acute HEV infection who presented with a generalized body rash and weakness in all four limbs. She was subsequently diagnosed with inflammatory myositis and treated successfully with steroids. We have reviewed relevant literature for a possible association. Myositis is a rare but known complication of HEV. If timely diagnosed and managed, there is a significant reduction in morbidity.

## Introduction

Hepatitis E virus (HEV) is a small, icosahedral, non-enveloped single-stranded RNA virus. The World Health Organization estimates that HEV causes 20 million new infections annually, with over 55,000 deaths [1]. It is prevalent worldwide, but predominantly seen in the developing countries. Transmission usually occurs through feco-oral route. It can also spread via blood transfusions and through mother-to-child transmission. Acute HEV infection is usually self-limiting and resolves within two to six weeks; however, it may lead to fulminant hepatic failure in some cases. The overall case-fatality rate is about 1% [2]. However it is 15-25% in case of pregnant females if the infection occurs in the third trimester [3]. The incubation period of HEV infection ranges from two to 10 weeks, with an average of five to six weeks [1]. There are four genotypes that are known to affect humans [4]. HEV genotype 1 has been reported as the dominant genotype in Pakistan with isolates Sar-55 (87-Pakistan-A), Abb-2B (88-Pakistan-2B), and 87-Pakistan-B [5]. Genotype 1 is also more common in Asia, Africa, and Latin America, while genotype 2 in Africa and Mexico, and genotypes 3 and 4 in the developed countries. Genotype 3 more commonly manifests with neurological symptoms [6].

Vast majority of patients with acute HEV are asymptomatic or have mild symptoms. In symptomatic patients, jaundice can be accompanied by malaise, anorexia, nausea, vomiting, abdominal pain, fever, and hepatomegaly. The extrahepatic manifestations of HEV may include but are not limited to blood dyscrasias, neurological disorders, acute glomerulonephritis, thyroiditis, and acute pancreatitis. A retrospective review of 106 patients with HEV was conducted in Devon and Cornwall, UK, which revealed that the extrahepatic manifestations were mostly hematological (58%), neurological (7.5%), or cardiac (0.94%). The neurological presentations included brachial neuritis, Guillain-Barré syndrome (GBS), peripheral neuropathy, neuromyopathy, and vestibular neuritis [7].

We present a case of severe myositis secondary to acute HEV infection. As yet, only three cases of hepatitis E-associated myositis have been reported worldwide to the best of our knowledge. However, no clear pathogenesis has yet been identified. These patients were treated with ribavirin and intravenous immunoglobulins (IVIG) along with steroids.

## Case presentation

A 27-year-old married female, right handed, functionally independent at home, presented to the emergency department (ED) at the Aga Khan University Hospital, Karachi, Pakistan, with the complaints of generalized body rash for three weeks, generalized weakness for two weeks, along with pain and tenderness in all limbs for four days. The patient was in her usual state of health three weeks back when she gradually developed a rash on her body starting from the neck and upper trunk, later on involving both upper limbs over the next one week. This rash was maculopapular, and associated with itching; however, it showed no discharge. For this, she took Unani medicines (constituents unknown) for a week with minimal improvement. In addition, she developed nausea and vomiting for which she visited a local hospital where she was tested positive for HEV and treated conservatively.

A week later, she developed generalized weakness that had a gradual onset and was progressive. She started having difficulty in combing her hair, rising up from sitting position, and in climbing stairs. Over the next two weeks, this weakness kept worsening due to which she was unable to walk without support. Four days before presenting to the ED, she developed pain and tenderness in all four limbs, especially in the bilateral shoulder and thigh muscles. The pain had a sudden onset, was gradually progressive, moderate to severe in intensity, and dull in character. It got aggravated with movement and temporarily responded to analgesics. There is no associated history of recent fever, weight loss, swallowing or breathing difficulty, numbness, tingling, bowel or bladder incontinence, oral ulcers, hair loss, or miscarriages. She is known to be allergic to penicillin among drugs and eggs among food.

**Figure 1:**
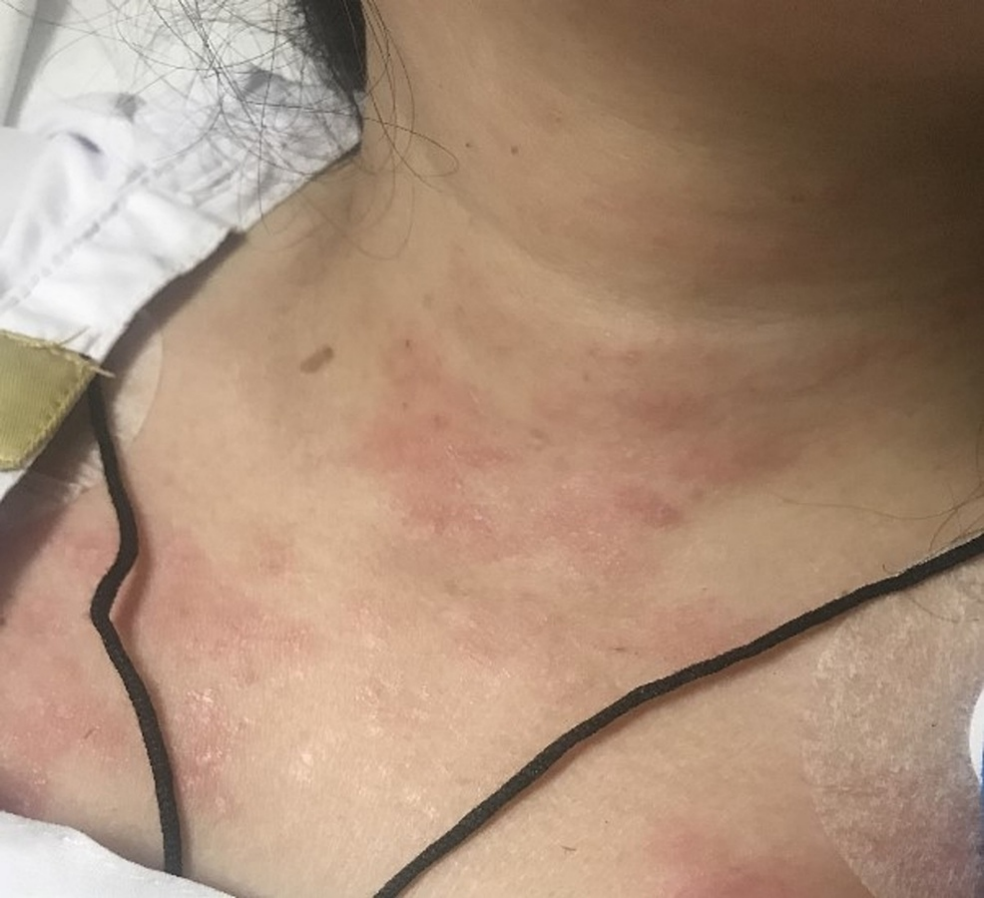
A maculopapular rash is visible over the neck and the upper trunk The picture was taken with the patient's consent.

On general physical examination, she was a young female of average height and built. She was awake, well oriented to time, person, and place, and cooperative during the examination. A maculopapular rash over the neck, upper trunk, and bilateral forearms was visible (Figure 1). There was no discharge from the rash. She did not have any rash or pigmentation around the eyes, face, knuckles, joint areas, or malleoli. No oral ulcers, joint pain, swelling, or tenderness was observed. There were no signs of anemia and jaundice. The rest of her systemic examination was unremarkable. On neurologic examination, the speech was normal, pupils were bilaterally equal, and reactive to light and accommodation. All cranial nerves were intact and there were no signs of meningeal irritation. On motor examination, the bulk, tone, and reflexes were normal. The muscle strength was assessed to be of Medical Research Council grade 2/5 power in the proximal muscles and 3/5 in distal muscles symmetrically. Her plantars showed bilateral flexor responses. The sensory, cerebellar, and gait examination was not possible due to severe pain and tenderness.

Initial blood work-up revealed mild hypochromic microcytic anemia and the liver function tests (LFTs) showed total bilirubin of 0.2 mg/dL, serum glutamic pyruvic transaminase (SGPT) of 729 U/L, and serum glutamic oxaloacetic transaminase (SGOT) of 604 U/L. Hepatitis E immunoglobulin M (IgM) antibodies were positive, while hepatitis B surface antigen and anti-HCV antibodies were negative. Creatine kinase (CK) was 13724 IU/L (normal range: 34-145), aldolase 29.5 U/L (normal 10-40), and thyroid-stimulating hormone was within normal limits. Serum beta human chorionic gonadotropin test was done to rule out pregnancy and it was negative. Nerve conduction studies were within normal limits, while electromyography revealed necrotizing myopathy affecting proximal muscles of the bilateral upper and lower extremities, which was severe in degree electrically. Autoimmune profile including antinuclear antibodies, anti-ds-DNA, anti-smooth muscle antibodies, antimitochondrial antibodies, and extractable nuclear antigen profile (anti-SMQN, anti-RO, anti-LA, anti-Sm, anti-Scl-70) were all negative. Lupus anticoagulant antibody was found to be weakly positive. Muscle biopsy was refused by the patient.

Dermatology opinion was taken for the skin rash and they diagnosed it as a fungal infection. Topical antifungals and oral antihistamines were advised for tinea incognito and pityriasis rosea after which the rashes started to improve. Gastroenterology team advised conservative management, close serial monitoring of LFTs, and ribavarin to be considered only if patient’s clinical status or LFTs worsen. Considering inflammatory/viral myositis, the patient was started on injection methylprednisolone 1000 mg IV once a day. Myositis autoantibodies panel was sent, which later came out to be negative. Patient was given five doses of IV inj. methylprednisolone followed by oral prednisolone 30 mg twice a day. On day 5 of admission, her liver enzymes showed an improving trend with SGPT and SGOT of 499 and 177 U/L, respectively, and a raised creatine phosphokinase of 2851 IU/L.

The patient was sent home on oral steroids and followed up in the clinic subsequently. After two months the skin rash had resolved completely and there was no muscle pain. Her muscle power improved to +4/5 in proximal muscles and 5/5 in distal muscles in all four extremities. Oral steroids were tapered off slowly over 12 weeks.

## Discussion

Viral myositis is a relatively common and self-limiting pathology probably more widespread than commonly thought. The clinical spectrum varies from bearable myalgia to life-threatening muscle weakness, rhabdomyolysis, and myoglobinuria. Influenza virus and enterovirus constitute the most common culprits [8]; however, the list is ever increasing with the recent inclusion of of SARS-CoV-2 [9]. Chronic hepatitis B and C viruses are frequently described to be associated with polymyositis, dermatomyositis, and inclusion body myositis in literature [10,11]; however, data related to hepatitis E-associated myopathy remain scarce.

The neurologic sequelae of HEV infection can involve the central or peripheral nervous system leading to acute transverse myelitis, acute meningoencephalitis, aseptic meningitis, neuralgic amyotrophy [12], pseudotumor cerebri, GBS [13], cranial nerve palsies, or, rarely, myositis [14]. As described below, diagnosis is usually confirmed through the detection of anti‐HEV IgM and IgG immunoglobulins, in appropriate clinical context with or without HEV RNA in serum and cerebrospinal fluid (CSF) along with exclusion of alternative differentials.

Although the possible mechanism of HEV‐associated neurological injury has been described sparingly in literature, the exact nature of injury to the muscles at molecular level is yet to be elucidated. The possible molecular pathogenesis of myositis in other viruses is indicative of either direct invasion, inflammatory cytokines released in response to viral infection [15], or immunological injury [16]. Correspondingly, ribavirin and interferons are the most widely used agents for the treatment of severe, complicated, and chronic HEV infections with resultant viral clearance despite majority acute HEV infections being managed only with supportive therapy [17].

The presence of HEV RNA in the CSF of patients with neurologic manifestations suggests that the local viral replication may occur in the central nervous system and lead to direct neuronal damage [18]. A Chinese study indicated that HEV is neuro-invasive and spinal cord tissue may be a better reservoir than the brain. The resultant tight junction defects, degenerative endothelial cells, and disorganized basal membrane layers cause increased permeability of the blood-brain barrier, which further facilitates the passive entry of the virions paracellularly [19]. In addition to direct viral damage, an indirect immune response that cross-reacts with axolemmal or Schwann cell antigens can lead to peripheral nervous system disorders, such as GBS in HEV-positive patients [18].

In 2012, Del Bello et al. reported the first case of GBS associated with severe necrotizing myositis that occurred in a 65-year-old liver-transplant patient in acute HEV infection phase. He presented with jaundice, deranged liver enzyme levels, positive serum HEV RNA, and positive anti-HEV IgG and IgM. Clinical examination and electrophysiological studies showed signs of both peripheral demyelinating polyradiculoneuropathy along with needle electromyography showing diffuse abnormal spontaneous activity in all four limbs. The CK level was 191,603 IU/L (normal <170 IU/L). CSF analysis showed elevated protein level of 64 mg/dL, no leukocytes, and no HEV RNA. Serum and CSF autoimmune and onco-neuronal antibodies were negative. A biopsy specimen of the left bicep brachial muscle showed myopathic changes, with a significant percentage of necrotic muscle fibers (10%) and some inflammatory elements. Patient was treated with IVIG (1 g/kg/day for two days) and ribavirin therapy according to creatinine clearance [400 mg/day (as per estimated glomerular filtration rate 40 mL/min)]. Serum HEV RNA became undetectable by day 15. Rehabilitation was continued and there was progressive recovery of mobility while ribavirin therapy was given for three months as initially scheduled [14].

As per the aforementioned retrospective review, Woolson et al. reported a patient with neuromyopathy, an 86‐year‐old man who presented with jaundice, nausea, and bilateral proximal arm and leg weakness. His LFTs were deranged, HEV IgG and IgM were positive, while CK was >21,000 IU/L. Electrophysiology studies and muscle biopsy confirmed the diagnosis of an inflammatory neuromyopathy. No immunosuppressive medicine or ribavarin was given in the case. Patient had long-lasting residual weakness [7].

Mengel et al. reported the case of a 57‐year‐old man with acute HEV infection that manifested as acute hepatitis, quadriparesis due to myositis, and renal failure. Electromyography revealed few complex repetitive discharges in proximal muscles with myopathic motor units and normal nerve conduction studies. Muscle MRI of the lower limb showed edema in proximal muscles (vastus lateralis, vastus medialis, and thigh adductors) with gadolinium enhancement. Muscle biopsy revealed scattered myofiber necrosis, and a diffuse mild lymphomonocytic infiltrate in the endomysium and perimysium. CSF analysis was normal without detectable HEV‐RNA. Cryoglobulins, porphyrins, and anti‐ganglioside and paraneoplastic antibodies in the serum as well as in the CSF were negative. Rhabdomyolysis resulted in renal failure with the subsequent need for dialysis. He was started on ribavirin (400 mg every 72 hours adapted to his glomerular filtration rate <15 mL/min), which resulted in a significant decrease in CK and liver enzymes, and it was continued for a total course of three months. The patient showed improvement, and on follow‐up at six months there was complete clinical recovery [20].

Though quadriparesis due to myositis unites all the above cases including ours, the female gender, younger age, presence of rash, and role of steroids make our case unique. The above-mentioned case reports uniformly describe old-age male patients (youngest being 57 years old) compared to our young female patient. None of the patients reported a skin rash as seen in our case. As it responded well to antifungal treatment, the rash most likely occurred due to a super-imposed fungal skin infection rather than HEV itself. Steroids have not been used in any of the above cases unlike ours. Two case reports described above stated rapid response to ribavarin, although there was a concomitant use of IVIG in one of the cases. No mortality has been described from the disease despite persistent morbidity in one case.

These previously reported cases show a similar temporal relationship between acute HEV infection and active inflammation in the muscles as in our case. In addition, the exclusion of other factors further suggests that this association is causal. Paucity of the available literature suggests HEV as a rare cause of severe myositis, however one that is treatable. While the vast majority of acute HEV infections are managed only with supportive therapy, ribavirin and immunosuppressants are the most widely used agents for the treatment of severe, complicated, and chronic HEV infections, which result in viral clearance [17].

## Conclusions

As is known, HEV is self-limiting and can rarely pose as a life-threatening disease. Cautious monitoring and active intervention are essential when clinical presentation extends beyond mild febrile icteric illness, malaise, and myalgias. HEV with its para-infectious complications has been treated successfully with ribavarin and immunosuppressive medications as per the available but limited literature. This study highlights that a unique case of severe myositis due to HEV that presented as quadriparesis and rash was treated successfully with steroids only.
